# Trends and seasonality in cause-specific mortality among children under 15 years in Guangzhou, China, 2008–2018

**DOI:** 10.1186/s12889-020-09189-0

**Published:** 2020-07-16

**Authors:** Xiao-Han Xu, Hang Dong, Li Li, Wen-Hui Liu, Guo-Zhen Lin, Chun-Quan Ou

**Affiliations:** 1 State Key Laboratory of Organ Failure Research, Department of Biostatistics, Guangdong Provincial Key Laboratory of Tropical Disease Research, School of Public Health, Southern Medical University, Guangzhou, 510515 China; 2 Guangzhou Center for Disease Control and Prevention, Guangzhou, 510440 Guangdong China

**Keywords:** Mortality, Seasonality, Children, China, Poisson regression model

## Abstract

**Background:**

This study analyzed the trends and seasonality in mortality among children aged 0–14 years in Guangzhou, China during 2008–2018. Understanding the epidemiology of this public health problem can guide policy development for children mortality prevention.

**Methods:**

A population-based epidemiological retrospective study was conducted. Seven thousand two hundred sixty-five individual data of children mortality were obtained from the Guangzhou Center for Disease Control and Prevention (CDC). The Poisson regression was used to quantify the annual average reduction rate and the difference in mortality rate between sex and age groups. Incidence ratio with 95% confidence interval (CI) was estimated to determine the temperaol variations in mortality by month, season, school term, day of the week and between holidays and other days.

**Results:**

Between 2008 and 2018, the children mortality rate in Guangzhou decreased from 54.0 to 34.3 per 100,000 children, with an annual reduction rate of 4.6% (95% CI: 1.1%–8.1%), especially the under-5 mortality rate decreased by 8.3% (95% CI: 4.8%–11.6%) per year. Decline trends varied by causes of death, even with an upward trend for the mortality of asphyxia and neurological diseases. The risk of death among males children was 1.33 times (95% CI: 1.20–1.47) of that of females. The distribution of causes of death differed by age group. Maternal and perinatal, congenital and pneumonia were the top three causes of death in infants and cancer accounted for 17% of deaths in children aged 1–14 years. Moreover, the injury-related mortality showed significant temporal variations with higher risk during the weekend. And there was a summer peak for drowning and a winter peak for asphyxia.

**Conclusions:**

Guangzhou has made considerable progress in reducing mortality over the last decade. The findings of characteristics of children mortality would provide important information for the development and implementation of integrated interventions targeted specific age groups and causes of death.

## Background

Prevention of child deaths is a key target for public health policy intervention at the national and international level, and child mortality is a widely used indicator of economic and social development [1]. In 2018, there were approximately 5.3 million deaths among children under 5 years of age and 0.9 million deaths among children aged 5–14 years globally [2]. The global under-5 mortality rate decreased by 59% from 93 deaths in 1990 to 39 deaths per 1000 live births in 2018, and this number declined from 53.7 to 8.6 deaths per 1000 live births in China between 1990 and 2018, with a reduction in the average annual rate of 6.5% [2]. However, the United Nations Inter-agency Group for Child Mortality Estimation (UN IGME) reported that the annual mortality reduction rate was lower among children aged 5–14 years compared with under-5 children since 2000 (2.7% vs 4.0%) [3]. The deaths at the age of 5–14 are predominantly from avoidable causes [3] but have largely been ignored by the global health community. The children at this stage of life undergo rapid developments which have major health consequences over the lifetime. It is suggested that some relevant global health targets, including the 2030 UN Sustainable Development Goals (SDGs), need to consider children up to age 15 years and not only those younger than 5 years [4]. This consideration would require better understanding the characteristics of mortality in children aged 5–14 years. However, most of the mortality data published were obtained from disease surveillance points or estimated from mathematical models rather than a whole population [3, 5, 6].

The Global Burden of Disease (GBD) Study 2016 reported that more than 3 million deaths in children were due to unintentional injury in 2015 and nearly 20% of them were under the age of 15 [7]. This amounts to 10% of the world’s children mortality in 2015 [7]. Approximately 90% of injury, mainly resulting from transport accidents, drowning, and asphyxia, is unintentional and can be prevented and controlled [8]. Previous studies suggested that children injury mortality might vary seasonally. Understanding the temporal patterns of children’s injury mortality can inform measures for injury prevention. Shinsugi et al. [9] observed a summer peak for transport accidents and drowning mortality and more deaths in winter for asphyxia in Japanese children. However, seldom did previous studies examine the differences in under-15 children mortality by day of the week, school term and between holidays and other days comprehensively.

The distribution of the causes of children death is influenced by the development of the social economy and healthcare [10]. Guangzhou, one of the fastest-growing economies in China, is currently experiencing the most rapid developments in the economy and healthcare. However, the children mortality rate in Guangzhou has not been comprehensively reported in the past decade and previous studies have focused only on injuries [11, 12]. This offers us an opportunity to determine the potential changes in the epidemiology of children deaths over time. This study aimed to examine under-15 children mortality by cause, year, sex and age group in Guangzhou during the period of 2008–2018 and to particularly elucidate the seasonal variations of injury-related deaths.

## Methods

### Data sources

In Guangzhou, the deaths are compulsorily registered and recorded in the death registration system operated by the Guangzhou Center for Disease Control and Prevention (CDC). And the mortality data were regularly cross-checked with the vital registration system operated by Public Security Bureau of Guangzhou Municipality in order to update some delayed or unreported death registration. We obtained individual data of deaths among residents of Guangzhou during 2008–2018, including sex, date of birth, date of death and cause of death. The annual number of permanent residents was collected from Public Security Bureau of Guangzhou Municipality where all permanent residents are registered. The data of children mortality were divided into four age groups: < 1 year, 1–4 years, 5–9 years and 10–14 years. Categorization of the causes of death followed the tenth version of International Categorization of Diseases (ICD-10). Causes of death were classified into four broad categories, including communicable, maternal, neonatal, and nutritional diseases (CMNN), non-communicable diseases (NCDs), injury and the ill-defined. Then, 24 subclasses were considered (see Additional file 1). The ill-defined proportion can be used as a measure of data quality [4].

### Statistical analysis

The proportions of deaths for four broad cause-of-death categories and for 10 leading causes of death by age group and sex are presented. The chi-square test was applied to compare the proportions of deaths for different causes of death between males and females for four age groups or betweent different age groups for males and females.

Annual mortality rate per 100,000 children was calculated for all children and specific age group and sex, using the corresponding annual number of permanent residents as the denominator. The average annual rate of reduction (AARR) in the mortality and 95% confidential interval (CI) were estimated separately for each age group and for males and females using quasi-Poisson regression analysis as follows:

$$\begin{aligned} \mathrm{Log}\left(\mathrm{E}\left[{\mathrm{Death}}_{ij\_age}\right]\right)&=\mathrm{Offset}\left(\mathrm{Log}\left({Pop}_{i}\right)\right)+{\beta}_{0j\_age}\\
&+{\beta}_{1j\_age}\ast {Year}_i+{\beta}_{2j\_age}\ast Agegroup,\\
&\mathrm{Log}\left(\mathrm{E}\left[{\mathrm{Death}}_{ik\_sex}\right]\right)=\mathrm{Offset}\left(\mathrm{Log}\left({Pop}_{i}\right)\right)+{\beta}_{0k\_sex}\\
&+{\beta}_{1k\_sex}\ast {Year}_i+{\beta}_{2k\_sex}\ast Sex,\\
&\mathrm{AARR}_{j\_age} = \left(1-\mathrm{exp}\left(\beta_{1j\_age}\right)\right) \times 100\%, j=1, 2, 3, 4,\\
&\mathrm{AARR}_{k\_age} = \left(1-\mathrm{exp}\left(\beta_{1k\_sex}\right)\right) \times 100\%, k=1, 2,
\end{aligned}$$

where *Death*_*ij_age*_ and *Death*_*ik_sex*_ are annual number of deaths for the jth age group and the kth sex group in the year *i*, respectively, *Pop*_*i*_ refers to the annual number of the population in the year *i*, *β*_1*j*_*age*_ and *β*_1*k*_*sex*_ are the corresponding estimates of the coefficients of variable *Year*, *Year* takes values of 1, 2, 3…11 from 2008 to 2018, *Agegroup* (i.e., < 1 year, 1–4 years, 5–9 years or 10–14 years) and *Sex* were defined as categorical variables.

Then, a quasi-Poisson regression model was fitted to annual number of deaths for different combinations of age group and sex to examine the statistical significance of the disparities in mortality rates by age group and sex, which was specified as follows:

$$\begin{aligned} \mathrm{Log}\left(\mathrm{E}\left[{\mathrm{Death}}_{ij\_age}\right]\right)&=\mathrm{Offset}\left(\mathrm{Log}\left({Pop}_{i}\right)\right)+{\beta}_{0j\_age}\\
&+{\beta}_{1j\_age}\ast {Year}_i+{\beta}_{2j\_age}\ast Agegroup,\\
&\mathrm{Log}\left(\mathrm{E}\left[{\mathrm{Death}}_{ik\_sex}\right]\right)=\mathrm{Offset}\left(\mathrm{Log}\left({Pop}_{i}\right)\right)+{\beta}_{0k\_sex}\\
&+{\beta}_{1k\_sex}\ast {Year}_i+{\beta}_{2k\_sex}\ast Sex,\\
&\mathrm{AARR}_{j\_age} = \left(1-\mathrm{exp}\left(\beta_{1j\_age}\right)\right) \times 100\%, j=1, 2, 3, 4,\\
&\mathrm{AARR}_{k\_age} = \left(1-\mathrm{exp}\left(\beta_{1k\_sex}\right)\right) \times 100\%, k=1, 2,
\end{aligned}$$

where *Death*_*i*_ is annual number of age- and sex-specific deaths in the year *i*.

Next, we conducted the stratified analysis for injury-related deaths by month, season, school term, day of the week and for holidays and other days. Four seasons were spring (March–May), summer (June–August), autumn (September–November) and winter (December–February). Based on the school calendar announced by Department of Education of Guangdong Province and Guangzhou Municipality, the school term was defined as the autumn term, winter vacation, spring term, and summer vacation. The incidence ratio (IR), estimated by *π*/*π*_0_, was used as a measure of variation in the number of injury deaths. The estimation of 95% CI was based on a normal approximation: $\pi /{\pi}_0\pm 1.96\sqrt{\pi \left(1-\pi \right)/n}/{\pi}_0$, where π and π_0_ are the observed and expected proportions of injury deaths in a specific time interval, respectively, and n is the total number of injury deaths for the entire study period. The expected proportion (π_0_) was estimated as the proportion of the number of days during a specific time interval among all study days, assuming a uniform distribution of daily deaths over time [9, 13, 14].

All statistical analyses were completed in R 3.6.1 (R Foundation for Statistical Computing). Statistical significance was set at *P* < 0.05.

## Results

**Fig. 1:**
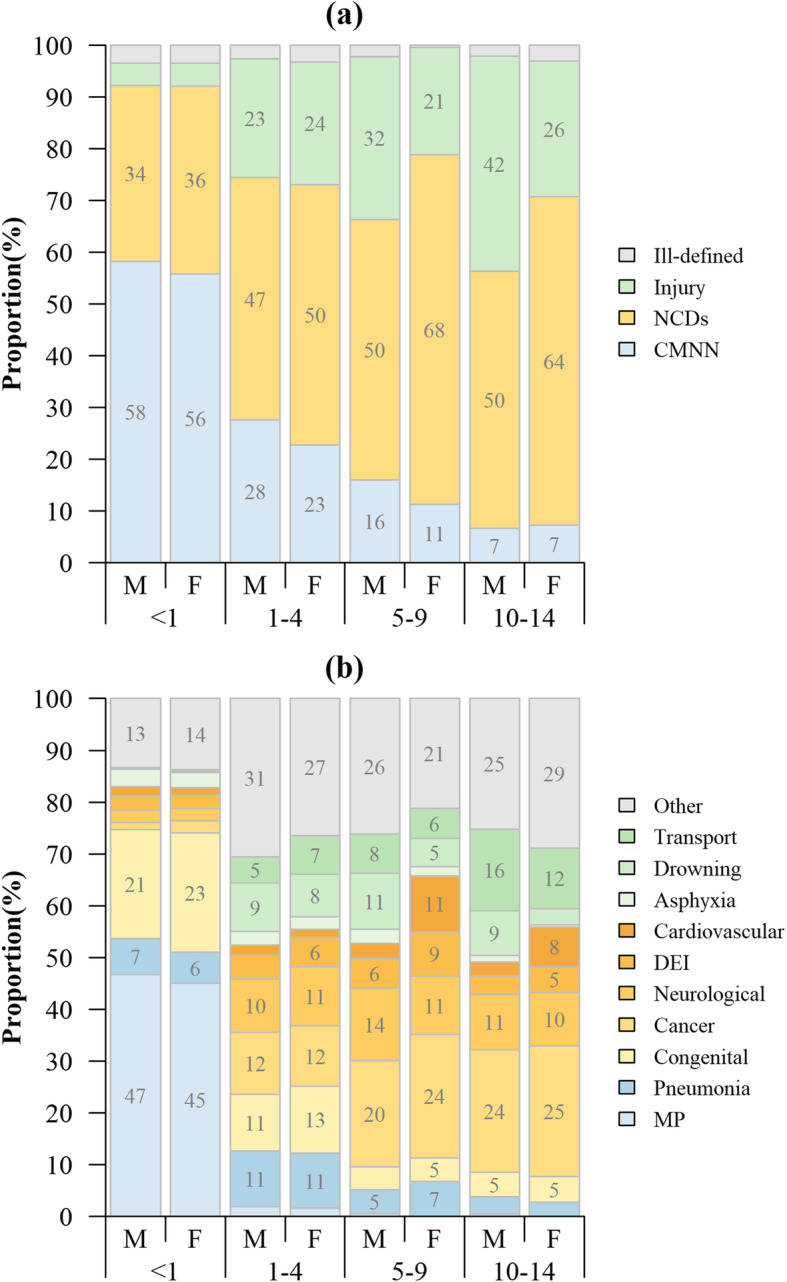
Proportions of death for different causes of death by age group and sex. (**a**) Proportions of deaths for four broad cause-of-death categories by age group and sex; (**b**) Proportions of deaths for 10 leading causes of death by age group and sex. NCDs: non-communicable diseases; CMNN: communicable, maternal, neonatal, and nutritional diseases; DEI: Diabetes, Endocrine, and immune disorders; MP: maternal and perinatal; M: male; F: female. Only the data for the causes whose corresponding percentages were greater than or equal to five are shown in the figure. In Panel (**b**), the blue, yellow and green parts represent the leading causes of CMNN, NCDs and injury, respectively

A total of 7265 children aged 0–14 years died during 2008–2018, among which 61% were males. There were 3101 (42.68%) CMNN deaths, 2990 (41.16%) NCDs deaths, 946 (13.02%) injury deaths and 228 (3.14%) ill-defined deaths. Figure 1 presents the proportions of deaths for four broad cause-of-death categories and for 10 leading causes of death by age group and sex. The broad cause-of-death categories were very similar between males and females under five, while for aged 5–9 and 10–14 years groups, males had a higher proportion of injury deaths and a lower proportion of NCDs deaths (*P* < 0.01) (see Additional file 2). The distribution of the 10 leading causes of death varied by age group (*P* < 0.01). The main causes of infant deaths were CMNN and NCDs, with nearly half from maternal and perinatal deaths and 21–23% from the congenital diseases. NCDs was a dominant cause in other age group. Specifically, the main causes of death in 1–4 years of children were pneumonia, congenital, cancer, neurological and drowning, each accounting for 8–13%. For children aged 5–14 years, the proportion of deaths due to cancer exceeded 20%, followed by the deaths from neurological diseases and transport injuries for both of males and females. The mortality rates of specific causes of death were shown in Additional file 3. Additionally, the total number of ill-defined deaths accounted for only 3.14% of all deaths with an average annual decline of 14.9%, indicating the improvement of mortality data quality in Guangzhou.

**Fig. 2:**
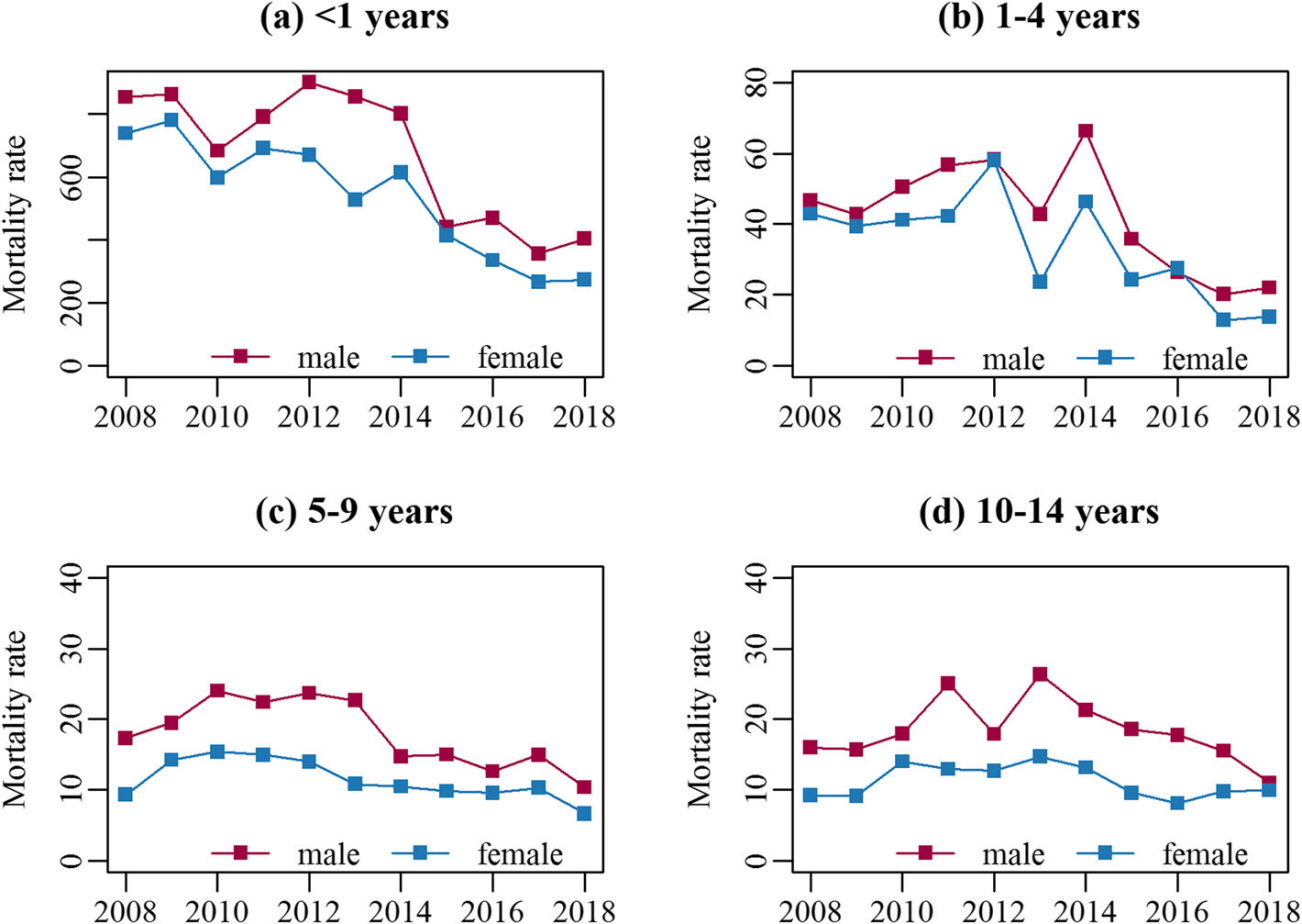
Trends of mortality rates (per 100,000 children) by age group and sex, 2008–2018. Panels (**a**-**d**) show the mortality for children aged < 1 year, 1–4 years, 5–9 years and 10–14 years, respectively

During the 11-year study period, the overall under-15 mortality rate declined from 54.0 per 100,000 children in 2008 to 34.3 per 100,000 children in 2018 (see Additional file 4), with an average annual reduction of 4.6% (95% CI: 1.1%–8.1%). The mortality rates of four causes-of-death categories declined by 2.9%–14.9% although the reduction for CMNN was statistically non-significant (Table 1). The decreasing trend in mortality was similar for males and females in all age groups (Fig. 2), while the mortality risk of males children was 1.33 times (95% CI: 1.20–1.47) as high as that of females (Table 2). Among four age groups, the infant death rate was the highest, with 342.1 per 100,000 children in 2018, in spite of an average annual reduction of 9.3% over the last 11 years. An average annual reduction of 8.9% was also observed for 1–4 years, while a high peak occurred in 2014. The mortality risks for infant and those aged 1–4 years were 40.94 times (95% CI: 34.22–49.37) and 2.61 times (95% CI: 2.13–3.21) of that for children aged 10–14 years. An average annual reduction in mortality for children aged 5–9 years was 5.7%, while there was no obvious decline in the mortality rate for those at the age of 10–14 years (Table 1, Fig. 2).

**Table 1:** Mortality rates per 100,000 children by age group and sex in Guangzhou, China

	All mortality in 2018	AARR%(95% CI)				
		All	CMNN	NCDs	Injury	Ill-defined
**Total**	34.3	4.6(1.1 to 8.1)	2.9(−0.9 to 6.4)	5.6(1.2 to 9.8)	4.9(0.8 to 8.9)	14.9(11.5 to 18.2)
**Age (years)**						
< 1	342.1	9.3(6.9 to 11.6)	8.1(5.7 to 10.3)	10.8(7.5 to 14.1)	4.6(− 2.1 to 10.9)	19.5(16.0 to 22.9)
1–4	18.2	8.9(4.9 to 12.8)	6.5(0.0 to 12.5)	10.4(5.9 to 14.7)	6.8(3.4 to 10.1)	22.2(15.3 to 29.0)
5–9	8.7	5.7(3.3 to 8.0)	3.4(−3.3 to 9.5)	4.3(0.9 to 7.5)	8.9(4.7 to 13.0)	18.5(−3.2 to 38.0)
10–14	10.5	1.4(−1.5 to 4.3)	1.2(−8.1 to 9.8)	−0.5(−4.4 to 3.3)	4.4(−0.6 to 9.2)	− 1.1(− 16.9 to 12.8)
**Sex**						
male	40.2	7.6(5.4 to 9.7)	6.8(4.3 to 9.3)	8.1(5.3 to 10.8)	6.1(2.9 to 9.2)	17.6(13.5 to 21.6)
female	27.5	9.3(7.3 to 11.2)	8.9(6.8 to 11.0)	9.5(6.6 to 12.4)	6.4(2.9 to 9.8)	20.2(13.0 to 27.3)

*CMNN* communicable, maternal, neonatal, and nutritional diseases, *NCDs* non-communicable diseases, *AARR* Average Annual Rate of Reduction, *CI* Confidence Interval

**Table 2:** Results of the Poisson regression model assessing the influential factors of children mortality

Variable	B (95% CI)	SE	RR (95%CI)	*P*
**Year**	−0.09(−0.10 to − 0.07)	0.01	0.92(0.90 to 0.93)	< 0.01
**Age (years)**				
10–14 (reference group)	–	–	–	–
< 1	3.71(3.53 to 3.90)	0.09	40.94(34.22 to 49.37)	< 0.01
1–4	0.96(0.76 to 1.17)	0.11	2.61(2.13 to 3.21)	< 0.01
5–9	0.03(−0.22 to 0.27)	0.12	1.03(0.80 to 1.31)	0.84
**Sex**				
Female (reference group)	–	–	–	–
Male	0.28(0.18 to 0.39)	0.05	1.33(1.20 to 1.47)	< 0.01

*CI* Confidence Interval, *SE* Standard Error, *RR* Relative Risk

**Fig. 3:**
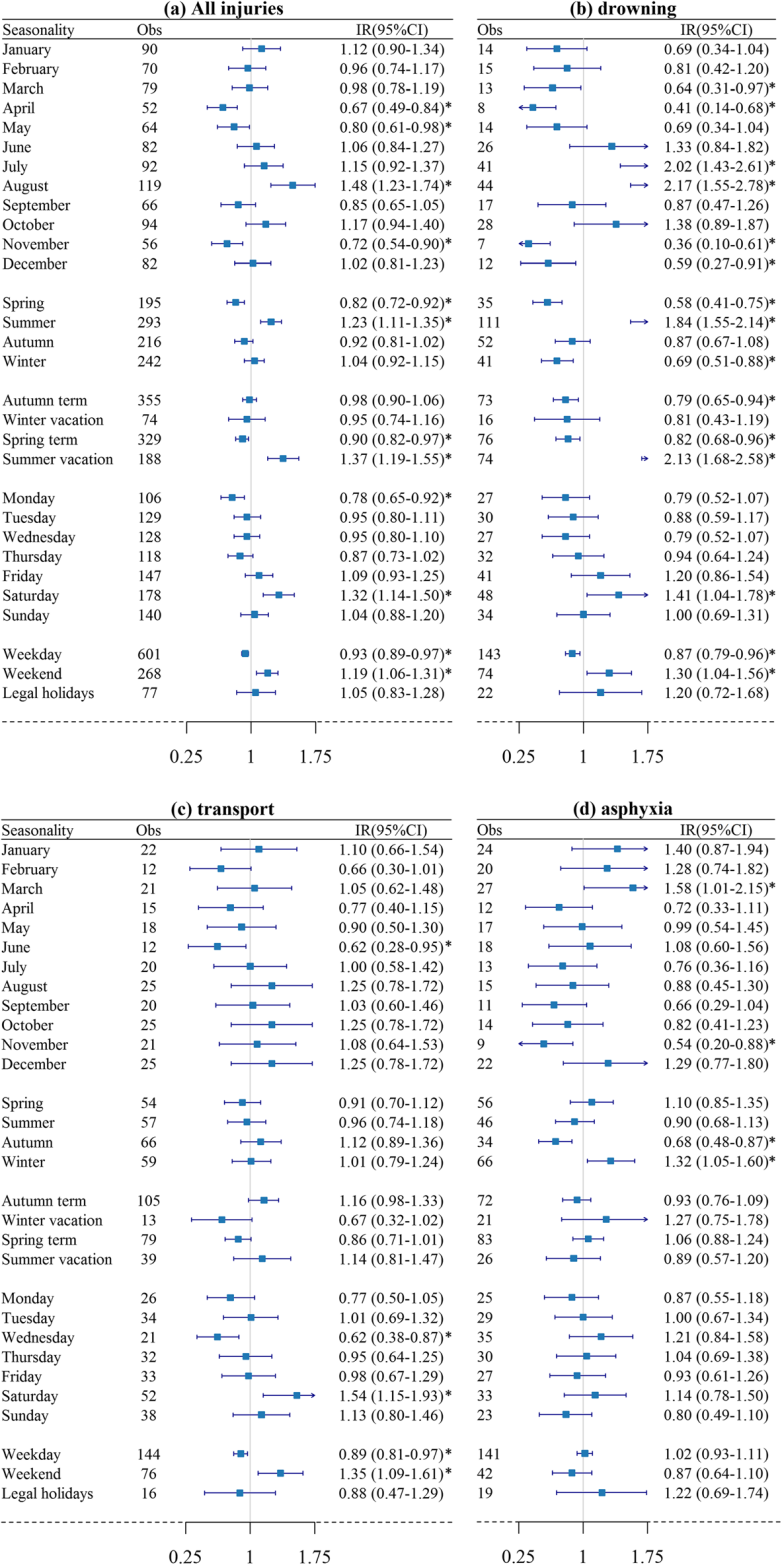
Incidence ratios and 95% confidence intervals of all injuries and three categories of injury-related death. * indicates statistically significant difference

Among 946 injury deaths, the three most common causes were drowning (25.26%), transport accident (24.95%) and asphyxia (21.35%). Figure 3 illustrates the temporal variations of injury deaths in multiple perspectives. Similar variations by day of the week were observed in all injuries, drowning, and transport accident deaths. The mortality rates were statistically significantly higher on weekends particularly on Saturday than weekdays. For all-cause injury, the mortality was significantly lower during spring (IR: 0.82, 95% CI: 0.72–0.92), and higher during summer (IR: 1.23, 95% CI: 1.11–1.35). An August peak (IR: 1.48, 95% CI: 1.23–1.74) and a lower peak in April, May, and November were observed. The risk of drowning death was double during July and August, accounting for 35.56% of drowning deaths. Consistently, a highest risk during summer vacation was observed (IR: 1.84, 95% CI: 1.55–2.14). In contrast, the risk was statistically significantly lower in March, April, November, and December. Transport accident deaths and asphyxia deaths presented less seasonal variations than drowning deaths. Neither showed variations in different school terms. The transport accident mortality was the lowest in June (IR: 0.62, 95% CI: 0.28–0.95). More asphyxia deaths happened in winter (IR: 1.32, 95% CI: 1.05–1.60), particularly in March (IR: 1.58, 95% CI: 1.01–2.15), while fewer in November and autumn.

## Discussion

This study assessed the mortality rates in children under 15 years in Guangzhou, China during 2008–2018. The infant mortality rates for males and females in Guangzhou were 9.0 and 6.7 per 1000 children, and 0.6 and 0.6 per 1000 children for those 1–4 years of age (see Additional file 4), which was relatively lower than the average level in China (infant mortality: 12.9 and 11.2 per 1000 live births; 1–4 years mortality: 2.1 and 1.9 per 1000 live births) [15]. Meanwhile, over the study period, the mortality rates declined from 1.8 to 0.8 per 1000 children for those aged 5–14 years, which was lower than the average mortality for this age group in China (4.3 to 2.4) [2]. The relatively low mortality rate could be attributable to the socioeconomic level of Guangzhou (ranked as the 7th Chinese city by Gross Domestic Product per capita in 2018) and the implementation of life-saving interventions, such as health education [16, 17]. In accordant with a multi-nation study [4], we found that the children mortality rates were greater in males than in females for all of the four causes-of-death categories. The possible reason was that females were generally more likely to develop early fetal lung maturity and were less engaged in more high risk-taking behaviors after the age of 1 year [6, 18]. And in many developed regions, the gradual disappearance of sex discrimination and the development of parental education have a female advantage in children mortality [19, 20].

In this study, a considerable decline was observed in the mortality rate of children aged under 5 years, which could benefit from the improvement of health services for children [21]. For those aged 5–14 years, reductions in mortality could be accelerated with efforts such as improving school meals, safety and healthy lifestyle education [3]. We found the mortality of children aged 10–14 years was higher than that of those 5–9 years of age and almost remained unchanged over the study period, probably because most of the public health interventions aiming at improving the health of children under 5 years may be beneficial for those 5–9 years of age, with smaller spillover effects to 10–14 age group [22].

Prevention interventions targeting specific age groups are required since the distribution of causes of death varied across age groups [23]. We found that the maternal and perinatal diseases accounted for the largest proportion of infant deaths, followed by congenital diseases. More attention should be paid to the primary and secondary prevention measures, including antenatal corticosteroids and kangaroo mother care, for preterm birth complications, a major part of maternal and perinatal diseases [24]. In addition, pregnancy at an appropriate age and the prenatal diagnosis are suggested to reduce the occurrence of congenital heart disease [23, 25, 26]. Our study indicated that the main causes of death for children aged 1–4 years were non-communicable diseases, while a national analysis reported injuries were the leading causes at this age group in China [17]. Different study periods or regional inequity in parental monitoring and child care could explain the disparity [27]. Cancer was the leading cause for children aged 5–14 years, most of which cannot be cured currently. This is why the mortality reduction was relatively low at this age group. More potentially curative treatments need to be explored in future studies [4].

Despite a continued reduction in children mortality, asphyxia-related mortality showed an overall upward trend throughout the study period (see Additional file 5). The key focus of preventative measures should be through training of the guardians on the prevention of ood obstruction and bed suffocation to reduce the infant deaths of accidental asphyxia [23, 28, 29]. The death rates from neurological cause among children aged 5–14 years also increased during the study period, especially in females [4] and cardiovascular diseases have been on the rise in recent years. Efforts, such as enhanced monitoring and prevention measures, are needed to mitigate the burden.

This study showed great seasonal variations of injury mortality. The incidence of drowning deaths was higher in summer vacation than in spring and winter. It could be due to the more frequent swimming in rivers and the sea in summer which increased the risk of possible death [9]. Therefore, prevention measures such as wearing a personal floatation device, strengthening the supervision of children’s activities, and teaching survival swimming and resuscitation skills should be implemented to reduce the drowning mortality [30]. We found that asphyxia mortality of showed a higher risk in winter than in other seasons. The winter peak could be attributable to the common mother-infant bed sharing in cold weather [9].

Our findings also revealed that the drowning and transport accident deaths occurred more frequently on weekends than weekdays. This is most likely because children spend more time outside, playing on the road or swimming in a river on weekends and therefore the possibilities of drowning and transport accident deaths increased [9]. Another possible reason is related to the popularity of shared bicycles in Guangzhou and the absence of mandatory requirements of drivers’ license or wearing helmet. The emergency system that deal with injuries should be strengthened during the weekends. World Health Organization (WHO) reported that children up to the age of 9 years are more likely to be accompanied by parents when they go out, while older children tend to be out more independently [27]. Therefore, for children under 9 years, the public education programs which enhance the parental monitoring might be an option for preventing injuries among younger children, meanwhile improving the child’s safety awareness is more important for children aged 10–14 years.

Our study has two strengths. First, we presented children mortality rates in Guangzhou based on the whole population data instead of sampling data, which avoided the problems of representativeness bias and low coverage rate. Second, we considered the temporal variations in cause-specific mortality rates at different time scales (e.g. month, school terms and day of the week), which have been seldom examined in previous studies.

Some limitations of our study should be mentioned. First, there were potential misclassification of the cause of death. We examined four major cases-of-death categories and top 10 specific causes and did not perform analysis for some uncommon causes by sex and age group due to the small number of deaths. The misclassification of cause of death should not be a serious issue when only broad causes of death were considered. Second, we cannot avoid the potential under-reporting of deaths particularly for infant deaths. It was estimated that the proportion of under-reporting was only approximately 0.31% during the study period using the empirical method proposed by Adair and Lopez [31]. Further, some individual and social factors, such as parental socioeconomic status, economy and growth environment, could influence the mortality risk. Further studies can be conducted to explore the impacts of these factors.

## Conclusions

The overall children mortality in Guangzhou declined between 2008 and 2018, with the most remarkable reduction of mortality occuring in children under-5 years of age, while the mortality remained stable among the children aged 10–14 years. The children mortality rate was higher among males and those under 5 years. Significant temporal variations were observed for injury-related mortality from drowning, transport accidents and asphyxia. The upward trends of mortality due to asphyxia, neurological and cardiovascular diseases highlight the importance of monitoring and management for the high-risk groups. Our findings provide important information for the development and implementation of interventions targeting specific causes, seasons and age groups to reduce children mortality.

## Supplementary information

**Additional file 1.** International Classification of Diseases (ICD) coding of the underlying specific causes of death.

**Additional file 2.** Comparisons of proportions of deaths for different causes of death categories.

**Additional file 3.** Leading causes of death for children by age group and sex in Guangzhou in 2018.

**Additional file 4.** Annual mortality rates by age group in Guangzhou, 2008–2018.

**Additional file 5.** Trends of age-standardized mortality rates by cause of death and sex in Guangzhou, 2008–2018.

## Data Availability

The data that support the findings of this study are available from Guangzhou Center for Disease Control and Prevention but restrictions apply to the availability of these data, and so the data are not publicly available. Permission can be requested by contacting Guangzhou Center for Disease Control and Prevention.

## Abbreviations

AARR: Average Annual Rate of Reduction
CDC: Center for Disease Control and Prevention
CI: Confidence Interval
CMNN: Communicable, Maternal, Neonatal, and Nutritional diseases
DEI: Diabetes, Endocrine, and Immune disorders
F: Female
GBD: Global Burden of Disease
ICD-10: The Tenth version of International Categorization of Diseases
IR: Incidence Ratio
M: Male
MP: Maternal and Perinatal
NCDs: Non-Communicable Diseases
RR: Relative Risk
SDGs: Sustainable Development Goals
UN IGME: United Nations Inter-agency Group for Child Mortality Estimation
WHO: World Health Organization
