# Supplementary material for: Trends and seasonality in cause-specific mortality among children under 15 years in Guangzhou, China, 2008–2018
Source: BMC Public Health. 2020 Jul 16;20:1117. doi: 10.1186/s12889-020-09189-0 (PMC7364532; doi:10.1186/s12889-020-09189-0)
Supplement: Supplementary file 1 — Additional file 1. International Classification of Diseases (ICD) coding of the underlying specific causes of death. [file 12889_2020_9189_MOESM1_ESM.docx]

Appendix table 1 International Classification of Diseases (ICD) coding of the underlying specific causes of death.

| Cause of death | ICD-10 range |
| --- | --- |
| **ⅠCommunicable and nutritional conditions** |  |
| Diarrhea | A02.8-A07, A08-A09.9 |
| Tuberculosis | A15-A19.9 |
| Meningitis / Encephalitis | A39-A39.9, A83-A86.4, A87-A87.9, G00-G00.9, G03-G03.9, G04-G05.8 |
| Nutritional deficiencies | D50-D53, E40-E46, E51-E64.0 |
| Pneumonia | J00-J06.9, J10-J18.9, J20-J22 |
| Maternal and perinatal | O00-O99.9, P00-P96.9 |
| CMNN other | A33-A35.0, A40-A79, A88.8, B00-B04, B05-B05.9, B06-B19.0, B20-B24, B25-B49, B92-B99, G02.0-J09 |
| **ⅡNon-communicable diseases** |  |
| Cancer | C00-C25.9, C30-C34.9, C37-C38.8, C40-C41.9, C43-C45.9, C47-C54.9, C56-C57.8, C64-C67.9, C69-C75.8, C81-C86.6, C88-C96.9, D00-D48.9 |
| Diabetes, Endocrine and immune disorders | D55-D64.8, D65-D89, E03-E07, E10-E14, E15-E16.9, E20-E34.9, E65-E88.9 |
| Neurological | F01-F99, G06-G98 |
| Cardiovascular | I00-I13, I20-I28, I38-I41.9, I42.1-I42.8, I43-I43.9, I44-I51.4, I60-I99 |
| Chronic respiratory disease | J30-J98.8 |
| Congenital | Q00-Q99.9 |
| NCDs other | C76-C80, C97-J09, D64.9-E14.1, H00-H61, I42.0-I42.9, I51.6-I51.9, L00-L98.4, M00-M99, N00-N64, K20-K92.9 |
| **ⅢInjuries** |  |
| Transport | V01-V99 |
| Fall | W00-W19.9 |
| Drowning | W65-W74.9 |
| Asphyxia | W75-W84.9 |
| Poisoning and fires | X00-X09.9, X40-X49.0 |
| Suicide | X60-X84.9 |
| Homicide | X85-Y09.9 |
| Undetermined intent | Y10-Y34.3 |
| Injury other | W20-W64, W85-W99, X10-X39, X50-X59.9, Y40-Y86 |
| **ⅣIll-defined** | R00-R99 |

CMNN, communicable, maternal, neonatal, and nutritional diseases; NCDs, non-communicable diseases.
