# Supplementary material for: Trends and seasonality in cause-specific mortality among children under 15 years in Guangzhou, China, 2008–2018
Source: BMC Public Health. 2020 Jul 16;20:1117. doi: 10.1186/s12889-020-09189-0 (PMC7364532; doi:10.1186/s12889-020-09189-0)
Supplement: Supplementary file 2 — Additional file 2. Comparisons of proportions of deaths for different causes of death categories. [file 12889_2020_9189_MOESM2_ESM.docx]

Appendix table 2 Comparisons of proportions of deaths for broad cause-of-death categories between males and females for different age groups.

| Age group | Categories | Number of death (%) | | χ2 | P |
| --- | --- | --- | --- | --- | --- |
|  |  | Male | Female |  |  |
| <1 year | CMNN | 1569 (58) | 1024 (56) | 2.98 | 0.40 |
|  | NCDs | 914 (34) | 667 (36) |  |  |
|  | Injury | 118 (4) | 80 (4) |  |  |
|  | Ill-defined | 94 (3) | 65 (4) |  |  |
| 1-4 years | CMNN | 242 (28) | 132 (23) | 4.51 | 0.21 |
|  | NCDs | 411 (47) | 292 (50) |  |  |
|  | Injury | 201 (23) | 138 (24) |  |  |
|  | Ill-defined | 24 (3) | 19 (3) |  |  |
| 5-9 years | CMNN | 65 (16) | 25 (11) | 18.76 | <0.01 |
|  | NCDs | 204 (50) | 150 (68) |  |  |
|  | Injury | 128 (32) | 46 (21) |  |  |
|  | Ill-defined | 9 (2) | 1 (0) |  |  |
| 10-14 years | CMNN | 28 (7) | 16 (7) | 15.54 | <0.01 |
|  | NCDs | 211 (50) | 141 (64) |  |  |
|  | Injury | 177 (42) | 58 (26) |  |  |
|  | Ill-defined | 9 (2) | 7 (3) |  |  |

CMNN: communicable, maternal, neonatal, and nutritional diseases; NCDs: non-communicable diseases.

Appendix table 3 Comparisons of proportions of deaths for 10 leading causes of death between different age groups for males and females.

| Sex | Categories | Number of death (%) | | | | χ2 | P |
| --- | --- | --- | --- | --- | --- | --- | --- |
|  |  | <1 year | 1-4 years | 5-9 years | 10-14 years |  |  |
| Male | MP | 1258(47) | 17(2) | 2(0) | 2(0) | 2148.5 | <0.01 |
|  | Pneumonia | 188(7) | 94(11) | 19(5) | 14(3) |  |  |
|  | Congenital | 568(21) | 96(11) | 18(4) | 20(5) |  |  |
|  | Cancer | 37(1) | 105(12) | 83(20) | 101(24) |  |  |
|  | Neurological | 64(2) | 91(10) | 57(14) | 45(11) |  |  |
|  | DEI | 74(3) | 39(4) | 23(6) | 15(4) |  |  |
|  | Cardiovascular | 48(2) | 18(2) | 12(3) | 12(3) |  |  |
|  | Asphyxia | 90(3) | 23(3) | 11(3) | 5(1) |  |  |
|  | Drowning | 5(0) | 82(9) | 44(11) | 37(9) |  |  |
|  | Transport | 5(0) | 45(5) | 31(8) | 67(16) |  |  |
|  | Other | 358(13) | 268(31) | 106(26) | 107(25) |  |  |
| Female | MP | 826(45) | 9(2) | 0(0) | 0(0) | 1310.8 | <0.01 |
|  | Pneumonia | 111(6) | 62(11) | 15(7) | 6(3) |  |  |
|  | Congenital | 422(23) | 75(13) | 10(5) | 11(5) |  |  |
|  | Cancer | 44(2) | 68(12) | 53(24) | 56(25) |  |  |
|  | Neurological | 44(2) | 66(11) | 25(11) | 23(10) |  |  |
|  | DEI | 46(3) | 33(6) | 19(9) | 11(5) |  |  |
|  | Cardiovascular | 27(1) | 9(2) | 24(11) | 17(8) |  |  |
|  | Asphyxia | 54(3) | 14(2) | 4(2) | 1(0) |  |  |
|  | Drowning | 4(0) | 48(8) | 12(5) | 7(3) |  |  |
|  | Transport | 6(0) | 43(7) | 13(6) | 26(12) |  |  |
|  | Other | 252(14) | 154(27) | 47(21) | 64(29) |  |  |

MP: maternal and perinatal; DEI: Diabetes, Endocrine, and immune disorders.
