# Supplementary material for: Trends and seasonality in cause-specific mortality among children under 15 years in Guangzhou, China, 2008–2018
Source: BMC Public Health. 2020 Jul 16;20:1117. doi: 10.1186/s12889-020-09189-0 (PMC7364532; doi:10.1186/s12889-020-09189-0)
Supplement: Supplementary file 3 — Additional file 3. Leading causes of death for children by age group and sex in Guangzhou in 2018. [file 12889_2020_9189_MOESM3_ESM.docx]

Appendix table 4 Leading causes of death for children by sex in Guangzhou in 2018.

|  | Male | | Female | | Total | |
| --- | --- | --- | --- | --- | --- | --- |
|  | Deaths (%) | Mortality rate per 100,000 | Deaths (%) | Mortality rate per 100,000 | Deaths (%) | Mortality rate per 100,000 |
| **CMNN** | 160(45%) | 18.1 | 85(40%) | 11.1 | 245(43%) | 14.8 |
| **NCDs** | 135(38%) | 15.2 | 90(43%) | 11.7 | 225(40%) | 13.6 |
| **Injuries** | 52(15%) | 5.9 | 29(14%) | 3.8 | 81(14%) | 4.9 |
| **Ill defined** | 9(3%) | 1.0 | 7(3%) | 0.9 | 16(3%) | 1.0 |
| **Leading causes** |  |  |  |  |  |  |
| Maternal and perinatal | 116(33%) | 13.1 | 68(32%) | 8.9 | 184(32%) | 11.1 |
| Congenital | 60(17%) | 6.8 | 35(17%) | 4.6 | 95(17%) | 5.8 |
| Pneumonia | 34(10%) | 3.8 | 9(4%) | 1.2 | 43(8%) | 2.6 |
| Cancer | 30(8%) | 3.4 | 12(6%) | 1.6 | 42(7%) | 2.5 |
| Neurological | 18(5%) | 2.0 | 15(7%) | 2.0 | 33(6%) | 2.0 |
| Asphyxia | 14(4%) | 1.6 | 10(5%) | 1.3 | 24(4%) | 1.5 |
| DEI | 9(3%) | 1.0 | 9(4%) | 1.2 | 18(3%) | 1.1 |
| Cardiovascular | 7(2%) | 0.8 | 9(4%) | 1.2 | 16(3%) | 1.0 |
| Drowning | 10(3%) | 1.1 | 6(3%) | 0.8 | 16(3%) | 1.0 |
| Transport | 9(3%) | 1.0 | 4(2%) | 0.5 | 13(2%) | 0.8 |
| **Total** | 356(100%) | 40.2 | 211(100%) | 27.5 | 567(100%) | 34.3 |

CMNN, communicable, maternal, neonatal, and nutritional diseases; NCDs, non-communicable diseases; DEI, Diabetes, Endocrine, and Immune disorders.

Appendix table 5 Leading causes of death for children by age group in Guangzhou in 2018.

|  | <1 years | | 1-4 years | | 5-9 years | | 10-14 years | |
| --- | --- | --- | --- | --- | --- | --- | --- | --- |
|  | Death (%) | Mortality | Death (%) | Mortality | Death (%) | Mortality | Death (%) | Mortality |
| **CMNN** | 213(57%) | 196.4 | 22(21%) | 3.8 | 7(15%) | 1.3 | 3(7%) | 0.7 |
| **NCDs** | 127(34%) | 117.1 | 43(41%) | 7.4 | 31(65%) | 5.6 | 24(56%) | 5.9 |
| **Injuries** | 21(6%) | 19.4 | 38(36%) | 6.6 | 9(19%) | 1.6 | 13(30%) | 3.2 |
| **Ill defined** | 10(3%) | 9.2 | 2(2%) | 0.3 | 1(2%) | 0.2 | 3(7%) | 0.7 |
| **Leading causes** |  |  |  |  |  |  |  |  |
| Maternal and perinatal | 179(48%) | 165.1 | 5(5%) | 0.9 | 0(0%) | 0.0 | 0(0%) | 0.0 |
| Congenital | 84(23%) | 77.5 | 8(8%) | 1.4 | 1(2%) | 0.2 | 2(5%) | 0.5 |
| Pneumonia | 26(7%) | 24.0 | 11(10%) | 1.9 | 5(10%) | 0.9 | 1(2%) | 0.2 |
| Cancer | 8(2%) | 7.4 | 14(13%) | 2.4 | 10(21%) | 1.8 | 10(23%) | 2.4 |
| Neurological | 10(3%) | 9.2 | 9(9%) | 1.6 | 8(17%) | 1.4 | 6(14%) | 1.5 |
| Asphyxia | 16(4%) | 14.8 | 7(7%) | 1.2 | 0(0%) | 0.0 | 1(2%) | 0.2 |
| DEI | 6(2%) | 5.5 | 8(8%) | 1.4 | 4(8%) | 0.7 | 0(0%) | 0.0 |
| Cardiovascular | 8(2%) | 7.4 | 0(0%) | 0.0 | 5(10%) | 0.9 | 3(7%) | 0.7 |
| Drowning | 1(0%) | 0.9 | 13(12%) | 2.2 | 1(2%) | 0.2 | 1(2%) | 0.2 |
| Transport | 1(0%) | 0.9 | 8(8%) | 1.4 | 0(0%) | 0.0 | 4(9%) | 1.0 |
| **Total** | 371(100%) | 342.1 | 105(100%) | 18.2 | 48(100%) | 8.7 | 43(100%) | 10.5 |

CMNN, communicable, maternal, neonatal, and nutritional diseases; NCDs, non-communicable diseases; DEI, Diabetes, Endocrine, and Immune disorders.
