# Supplementary material for: Trends and seasonality in cause-specific mortality among children under 15 years in Guangzhou, China, 2008–2018
Source: BMC Public Health. 2020 Jul 16;20:1117. doi: 10.1186/s12889-020-09189-0 (PMC7364532; doi:10.1186/s12889-020-09189-0)
Supplement: Supplementary file 4 — Additional file 4. Annual mortality rates by age group in Guangzhou, 2008–2018. [file 12889_2020_9189_MOESM4_ESM.docx]

Appendix table 6 Annual mortality rates by age group in Guangzhou, 2008-2018.

|  | All mortality (deaths per 100,000 children) | Sex-specific mortality for children under 1 year (death per 1,000 children) | | Sex-specific mortality for children aged 1-4 years (death per 1,000 children) | | Probability of dying among children  aged 5-14 years (deaths per 1,000 children aged 5 years) |
| --- | --- | --- | --- | --- | --- | --- |
|  |  |  |  |  |  |  |
|  |  | Male | Female | Male | Female |  |
| 2008 | 54.0 | 8.5 | 7.4 | 0.5 | 0.4 | 1.8 |
| 2009 | 55.9 | 8.6 | 7.8 | 0.4 | 0.4 | 1.7 |
| 2010 | 59.3 | 6.8 | 6.0 | 0.5 | 0.4 | 2.0 |
| 2011 | 66.0 | 7.9 | 6.9 | 0.6 | 0.4 | 2.0 |
| 2012 | 74.8 | 9.0 | 6.7 | 0.6 | 0.6 | 1.6 |
| 2013 | 60.9 | 8.5 | 5.3 | 0.4 | 0.2 | 1.7 |
| 2014 | 68.9 | 8.0 | 6.2 | 0.7 | 0.5 | 1.4 |
| 2015 | 46.3 | 4.4 | 4.1 | 0.4 | 0.2 | 1.1 |
| 2016 | 44.7 | 4.7 | 3.3 | 0.3 | 0.3 | 1.0 |
| 2017 | 39.3 | 3.6 | 2.7 | 0.2 | 0.1 | 0.9 |
| 2018 | 34.3 | 4.0 | 2.7 | 0.2 | 0.1 | 0.8 |
