# Supplementary material for: Trends and seasonality in cause-specific mortality among children under 15 years in Guangzhou, China, 2008–2018
Source: BMC Public Health. 2020 Jul 16;20:1117. doi: 10.1186/s12889-020-09189-0 (PMC7364532; doi:10.1186/s12889-020-09189-0)
Supplement: Supplementary file 5 — Additional file 5. Trends of age-standardized mortality rates by cause of death and sex in Guangzhou, 2008–2018. [file 12889_2020_9189_MOESM5_ESM.docx]

Appendix figure1 Trends of age-standardized mortality rates by cause of death and sex in Guangzhou, 2008-2018.


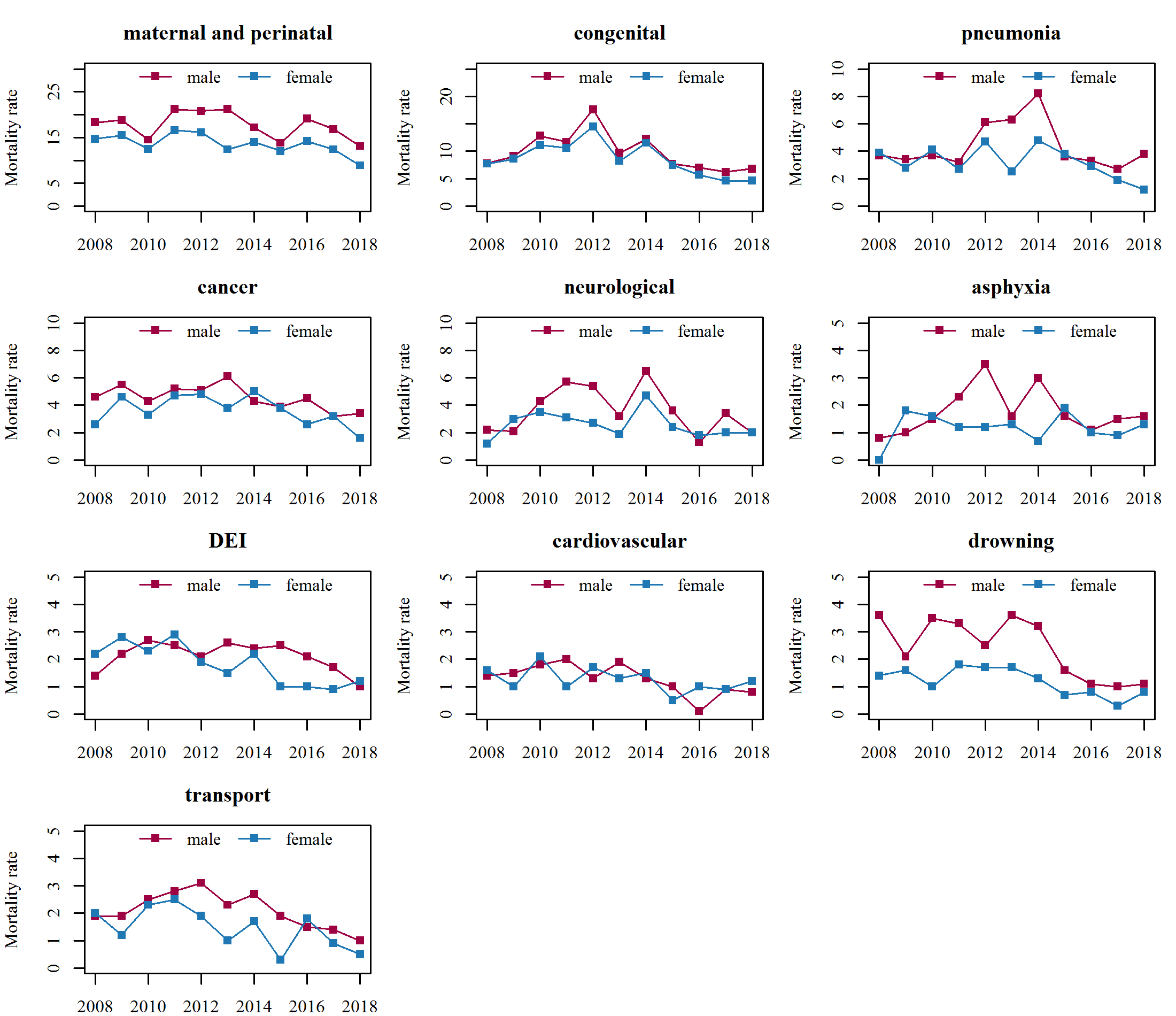


DEI: Diabetes, Endocrine, and immune disorders.
